# “There is nothing better than participating in this study”: Living the PAPAartis cardiovascular randomised controlled trial^☆^

**DOI:** 10.1016/j.conctc.2022.100987

**Published:** 2022-09-03

**Authors:** Nuria Romo-Avilés, Johanna Fröhlich Zapata, Alena Keuneke, David Petroff, Christian D. Etz, David Epstein

**Affiliations:** a Department of Social Anthropology, University of Granada, Spain; b Medical Anthropologist, Medical Anthropology Research Center, Berlin, Germany; c Clinical Trial Centre, University of Leipzig, Germany; d University Department for Cardiac Surgery, Leipzig Heart Center, Leipzig, Germany; e Department of Applied Economics, University of Granada, Spain

## Abstract

Qualitative research can bring new dimensions of understanding decision-making process in clinical trials. Participating in a randomized clinical trial requires patients to accept complex information and make decisions in a context of uncertainty. It becomes especially complicated in the case of serious diseases in which the treatment itself implies unknown risks. This study examines these issues in the context of the PAPAartis randomized clinical trial, which aims to prevent spinal cord injuries that can occur as an adverse event following complex surgical repair of thoracoabdominal aneurysm. In this study, we accessed a group of 16 patients participating in the trial and, through in-depth interviews, sought to understand the decision-making process when taking part in the trial and their experience of it. Our results showed that patients participated for different reasons: due to trust in doctors, the hope of having a better treatment or for altruistic and collaborative reasons with science. Many patients felt they did not fully understand the extraneous information provided about the study and the complex nature of the procedure. Avoidance of paraplegia played a fundamental role in the decision to participate in this trial. Family support and the socioeconomic conditions of the patients influenced the recovery process after surgery.

## 1 Introduction

Traditionally, clinical studies focused on measuring objective parameters and largely ignored the patient's experience. More recently, the patient's perspective has grown in importance, acknowledging that fact that a clinical trial aims primarily at improved patient care, and considering it can significantly enhance the conceptualization and implementation of research. Embedding a qualitative study in clinical investigation is now increasingly encouraged, even required, by some research funding bodies. Using qualitative research methods alongside essentially quantitative trials can enhance the design, conduct, and findings [1]). It is possible to obtain insights about patients' understandings and feelings, as well as human behaviour and organisational factors that are difficult to capture in quantitative research [2]. Often, effectiveness in terms of healing depends upon adherence [3] cit. a Mozygemba et al., 2016) or acceptability of the intervention [4]. Psychological and social factors are often modifiers, the “soft” concerns that amplify either the distress or the healing process [5]). Qualitative studies can highlight the role of informal carers and how they can be involved in a rehabilitation programme [6] or otherwise support the patient [7]. By capturing patients' voices and perceptions of quality of care, particularly distressing experiences might be avoided in future [8].

The highest quality of clinical evidence is often considered to be the randomised controlled trial (RCT). Evidence from RCTs is an essential requirement for the approval of medicines and other health technologies by regulatory bodies and pre-requisites for their adoption by health services. However, their operation, concepts and rituals are often unknown to the general public. Patients must actively choose to participate in a clinical study and the central ethical concern and challenge for the investigator is “informed consent”. This means ensuring that patients understand the scientific nature of the trial. In particular, they will be randomly chosen to undergo either the experimental treatment under investigation, or a control treatment, often “usual care”. However, people assign different meanings to written information that clinicians and investigators assume to be objective and unambiguous. While most state that in general they are capable of conceiving and processing the information given, others struggle to comprehend the meaning and purpose of concepts such as randomisation and double blinding [9]. Indeed, they may even find them threatening to their ideas of medical care [10]. For some patients, such decision-making seems to be an immediate reflex rather than reflection on given information [11].

Clinical investigators should only conduct a trial when there is “collective equipoise”, that is, before the study begins there is no conclusive evidence demonstrating that one treatment is superior to the other. From the perspective of the patient, however, the literature identifies several possible motives and barriers for choosing to participate. One strong theme is the altruistic motivation to “serve science” [12]. Some patients express a feeling of empowerment [13]. Trust and belief in the expertise of the clinician-investigator plays an important role [11]. People may expect better care in a clinical study than in routine practice, regardless of the assigned treatment [12]. They express a feeling of “being taken care of” [13]. Indeed, as [12] concluded, these two themes are intertwined. Participants do not join a trial just because of altruistic motives, they also want to benefit individually.

The dual nature of the clinical investigator (both researcher and physician) is also reflected in the way patients perceive their role [14]. suggested that some participants saw themselves as “volunteers”, others as “real patients”. This difference in perception of their own “identity” seemed to be reflected in their actions during participation in the trial. Patients who tended to see themselves as volunteers might not make decisions about a specific treatment as much as participants who see themselves more as a patient - and tend to accept unforeseen results of the trial. They hypothesize that the patient's identity within a trial might even alter the patient's behaviour [14].

This paper reports a qualitative study embedded in an RCT that is studying a very high risk procedure to repair thoracoabdominal aortic aneurysms (TAAA). An aneurysm is a weakness of a blood vessel and TAAA is a very rare condition that is among the most challenging types of aneurysms to treat. Patients with TAAA are at risk of the sudden rupture of the aneurysm at any moment, which is usually fatal, and are often advised by their clinician to consider invasive complex treatment to repair the aneurysm. There are several TAAA repair techniques available, including endovascular TAAA repair (TEVAR) or open repair. Although these procedures are only carried out in highly specialised centres by the most expert clinical teams, they nevertheless carry a substantial risk of complications, including the risk of leaving the patient paraplegic.

Recently, a promising new technique has been developed, known as the staged-repair concept or MIS^2^ACE (minimally invasive staged segmental artery coil embolisation) with the aim of providing protection from paraplegia [[15], [16], [17]]. MIS^2^ACE is an additional procedure conducted some time before the main aneurysm repair and, paradoxically, it works by deliberately occluding arteries, which then induces arteriogenesis so that the collateral network can provide a robust blood supply to the spinal cord after TAAA repair. The PAPAartis clinical trial was set up to compare the new intervention (that is, MIS^2^ACE followed by TAAA repair) to standard care (that is, TAAA repair without MIS^2^ACE) [18]. The clinical trial continues to recruit patients from 27 sites throughout Europe. This paper focuses on qualitative fieldwork conducted with patients recruited from the German sites with the objective of understanding the different assessments that patients have about the impact of the interventions that have been performed on them and the consequences for their health biography. Our research question is: What has been the experience of patients in the Papa-Artis clinical trial?

## 2 Methods

### 2.1 Procedure for setting up and conducting interviews

In-depth interviews were used to obtain a non-intentional sample. The interviews used a prompt guide with broad topic areas, but the emphasis was on encouraging patients to discuss their own perspectives freely and allow them to raise issues that were important to them. Once the necessary consent process in the qualitative work requested by the nursing staff of the trial had been clarified, the key informant nurse at Universitätsklinik Leipzig provided the qualitative study researchers with an encrypted list of patients at the different centres who could be interviewed, and their doctors and study nurses. In some centres, we were asked to write a letter to the patients to introduce ourselves, in other cases the study nurses would introduce our work to the patients preparing them to be contacted by us.

The researcher tested the interview procedures with the first two patients. After these interviews, we reviewed the prompt guide in the light of the initial experiences and members of the qualitative research visited the trial centres again in order to facilitate contact and access with further patients. From here, we began a continuous process of face-to-face interviews at a location of the patient's choice, for example, in the private homes of the participants or in the cafeterias of the hospitals with the patients, as well as conducting meetings with study nurses at the different centres.

The safety, comfort and convenience of the patient was of primary importance. The study nurses only gave the research team the details of patients who had recovered from the surgery. In one case, the patient told the researcher that she had partially lost her voice following the surgery and so an interview was not possible. All other proposed interviews were able to go ahead. Patients were aware they could terminate at any time.

Face-to-face interviews began in late 2019. The interruption of the COVID-19 pandemic in March 2020 required us to adapt the study plan while ensuring the quality of research was maintained (Tremblay S. et al., 2021). All subsequent communication and interviews with patients were carried out by telephone. The research procedure was explained and the consent of the patient was obtained in an initial call, and a time was scheduled for the full interview. The telephone interview was carried out following as far as possible the same protocol that had been applied in the face-to-face interviews.The positive aspect of these phone interviews, that can feel less personal, is that we felt like patients were sometimes more open to speaking about something in detail. On the other hand, in person interviews would have probably been a better way to study the patients' social background, their illness narrative, since they began in their homes and would have allowed us to participate in the context of life and suffering of the participants.

Table 1 shows the characteristics of the participants (N = 16) in our qualitative trial. Half of the participants were women (n = 8) and men (n = 8). Eight received the MIS^2^ACE intervention, but a ninth participant allocated to this group died before the procedure could be carried out. The average age of the participants was 69 years (range 43–80, 1 not stated, access to the trial database was excluded to maintain anonymity). At the time of the interview, 8 were retired, 2 on sick leave and 6 did not state their employment status.

**Table 1:** Participants in the study.

	Men (N = 8)	Women (N = 8)	Total (N = 16)
**Age in years,** range (mean) Not stated by participant	43–80 (∼68) 1	58–79 (∼70)	43–80 (∼69) 1
**Randomised treatment** MIS^2^ACE Control group	5 3	4 4	9 7
**Occupation**	Toxicologist, farmer, civil engineer, bank employee, administration, not stated (2)	Podologist, factory worker, public administration, material management, saleswomen (2) hairdresser, not stated (1)	
**Current working situation** Retired Sick leave Not stated			
	4	4	8
	1	1	2
	3	3	6
**Civil status** Married Divorced Widowed Single Not stated			
	6	4	10
	1	1	2
	0	2	2
	0	1	1
	1	0	1

### 2.2 Data analysis

The average time of the interviews was 60 min. All the interviews were recorded and transcribed literally in German, and subsequently post edited and translated into English.The research team consisted of 4 people, each of whom contributed to producing an accurate translation and analysis. The interviews were conducted and recorded in German by a native German speaker with experience of qualitative study in a medical setting. The recordings were then transcribed into German by a second investigator, also a native German speaker. This step was facilitated by specialist AI transcription software (NVIVO-TRANSCRIPTION). The same researcher (who also has a very high level of English) then translated the transcripts into English, aided by the other researchers, including a native English speaker. This was the text that was analysed in NVIVO. Those parts of the interviews in which there could be some doubt were specifically reviewed to avoid loss of meaning, holding meetings to discuss some of these aspects between all the researchers. One of the challenges noted by the two German speakers was that some of the interviewees spoke in strong regional dialects, and certain words and expressions were unfamiliar. However, these instances were very infrequent and could be resolved by examining the context in which the interviewer was speaking.

A summative content analysis was carried out. After the first general reading of the transcripts, members of the team undertook the first identification of codes and categories. This initial comparison of categories made it possible to agree on criteria for the process of codification and thematic units of interest. Subsequently, another member of the team collated the categories developed. The end point of the process of data collection was determined following the principle of theoretical saturation [19]. defines saturation as ‘the point in coding when you find that no new codes occur in the data. There are mounting instances of the same codes, but no new ones’. We operationalized saturation in a way that is consistent with the research question and the theoretical position and analytic framework adopted [20]. Following the principles of Grounded Theory, we attained a diverse sample in the categories stated and we incorporated them into the analysis. Grounded theory was applied in the whole research process because collecting, coding and analysis of data took part from the beginning of the research process. We try to be theoretical sensitive, giving meaning to data, understanding what the data says, and being able to separate out what is relevant and what is not. We develop a theory that is grounded using theoretical sampling to guide the researcher as data were collected. It helped facilitate the development of theory as it emerged. Saturation point was reached when the team agreed that the codification was no longer providing significant information in the analysis process. This triangulation process made it possible to test the level of consistency and solve discrepancies. After the codification, the most significant units of analysis were extracted and the interrelations between the different themes were identified. The entire analytical process was developed with the aid of the QSR NVivo 12 program.

### 2.3 Rigour and quality

Quality and rigor have been maintained in data collection and analysis. First, we develop and refine a primary research question. Our conceptual framework comes from medical anthropology from where the research question was formulated and the techniques to be used to obtain data were decided. Having a strong conceptual framework facilitated the choice of an appropriate research technique for personal interviews despite the impact of the pandemic on fieldwork. Another aspect to maintain rigor and quality has been to define and establish the sample and for this we have had the collaboration of the clinical team. We have maintained rigor on the basis that the data have been obtained inductively, within the framework of the clinical trial, but from the participants themselves towards the search for results, as is usually done in qualitative research. The reflexivity and teamwork that has been continuous and online throughout the pandemic has helped control possible pathways and maintain the necessary rigor.

### 2.4 Ethical considerations

The study was approved by the lead Ethics Committee from the University of Leipzig (435/17-ek) and the Ethics Committees at each of the sites. The names and sites of participants in the results have been changed to protect anonymity.

## 3 Results

The content analysis of the interviews has been grouped into four broad themes, summarised in Table 2:

1. The patients' hopes and expectations of choosing to participate in the PAPAartis clinical trial.
2. The benefit-risk balance of choosing to be treated for TAAA, including fear of paraplegia.
3. Impact on family life of illness and treatment.
4. Impact of COVID-19 on the recovery process.

**Table 2:** Summary of the main results.

Hopes and expectations of choosing to participate in the PAPAartis RCT	o Understanding an RCT and informed consent o Trust in the investigator - clinician o Perception that care would be better within a clinical study o Belief that participating in the study would mean getting the new intervention o Help science or moral duty
The benefit-risk balance of choosing to be treated, and probability of paraplegia	o Fear about risk of paraplegia o Disappointment about being allocated to control treatment o The value of hope
Family life	o Burden of illness on the family o Desire for independence and control o Being closer as a family
Treatment and recovery during the pandemic	o Undergoing major surgery during a pandemic o Vulnerability to COVID-19 o Cancelled appointments

### 3.1 Hopes and expectations of choosing to participate in the PAPAartis randomised controlled trial

1.an Understanding a randomised clinical trial and giving informed consent to participate

The patients who participated in this trial received information about their operation and the different possibilities and risks. The information pertaining to the trial has to be approved by the Ethics Committees, and the requirements of “Good Clinical Practice” lead to very lengthy material that contains long passages on topics of little interest to many participants, such as those pertaining to the European “General Data Protection Regulation”. In some interviewees, the medical recommendations were important, in others, patients indicated that they did not understand the procedure well at all.

Mrs. Krüger: *Yes, but that’s 9 out of 21 pages.. (…) until you understood that all. I still think that’s an imposition!*

Interviewer: *You have to explain more precisely. It’s an imposition because..?*

Mrs. Krüger: *because there is too much*

Interviewer: *there is too much information?*

Mrs. Krüger: *yeah what … it starts … why they undertake the examination? Blum blum blum, yeah I think (…) I am able to read that all but to understand, That’s another thing..*

(Mrs. Krüger, 77, receives the new treatment)

1.b. Trust in the investigator-clinician

Medical information has to be trustworthy and to encourage the patient by offering expectations of good treatment and good results. Besides the descriptions through the physicians, patients' conception of “how a trial is” or “how it could be” plays an important role in the decision-making.

Interviewer: *Could you please describe this decision-making process in more detail?*

Mr. Koch: *So. I had not given myself any time to think about it. I immediately gave Dr. Klaus my okay to do so. Directly on the phone.*

Interviewer: *Could you tell me what you thought on the phone at that moment?*

Mr. Koch: *Well, I was glad that somebody was trying so hard for me. So. I felt cared for. I thought that if you participated in it, then:****nothing better could happen to you.****Well, no fears came up, so that […] if this is what you want to know. On the contrary, it was all good.*

(Mr. Koch, 63, receives the new treatment)

Not all the cases develop the same confidence in the clinical process. For example, in the case of Mrs. Weber, it is her own decision that appears first, surprising her distance from clinical information or from the search for any type of medical information.

Mrs. Weber: *To be honest, I don’t know how to say it. But if you participate in a trial,****I guess, you get more attention or, or they try things out which they normally don’t try out or something. I told myself, it can’t be bad. It only can have advantages****. I copied the trial [documents] and brought it to my family doctor’s.*

… and said to let him know in what I am participating, and he told me it was nothing bad, it was nothing wrong. And first of all, it was for other people or you served science and you had to somehow.., that you advanced or to have new findings. And I told myself “It can only be good for me”. I really have to say.

(Mrs. Weber, 58, MIS^2^ACE group)

1.c Perception that clinical care would be better in an RCT than in routine practice

As we can see in the previous discourses, the decision to participate in the trial could come from medical information or from one's own intuition, always guided by the interest in obtaining better medical care and some advantages in the clinical intervention.

Mr. Richter: *yeah well that was certainly.. that was certainly good, ehhh yeah ehh they called and asked that there was a kind of trial ..and if I’d like to participate … and basically I must say [admit] honestly what you always hear.. is..****if you are in a trial that you get a better [medical] attendance****than.. a simple, normal patient.. and then they all said simply: “yes do it!” …*

and so I did …

(Mr. Richter, 68, control group)

In some of the cases the families' discourses play an important part in the decision to take part in the trial. Some patients and their family members think that participating in a trial is something good and carries less personal risk than routine practice.

Mrs. Klein: *I have talked about it with my children and with my husband.*

And they all had the opinion that that. That I could do that. That would be a good thing and […] **more security for me.**

(Mrs. Klein, MIS^2^ACE group)

As we can observe in the discourses, trust in the clinical staff, “good feelings” and especially believing in their expertise were essential for participating.

Interviewer: *You say that with an expression of pride?*

Mrs. Müller: *yeah because it’s somehow like that..*

Interviewer: *Yeah?*

Mrs. Müller: ***I feel good here, well in good hands..here***

(Mrs. Müller, 67, control group)

1.d Belief that participating in the study would mean getting the new intervention

One patient stated that a reason for participating in the clinical study was to obtain the new intervention. However, in principle, the new intervention is not available in normal practice, and patients who participate only have a 50% chance of obtaining the new intervention. In fact, this patient was randomly assigned to the control group.

Mrs. Schröder: *Yes. With the new method. (…) Yes, yes, and then I also wanted to take part in the study, because that might be a bit more positive,****because you have these pre-operations or interventions and that's why I signed up for the study.***

(Mrs. Schröder, 72, control group)

1.e. Helping science through participation in a clinical study or a moral duty to participate

Some patients are quite convinced about participating in medical trials, not only because of their own safety, welfare and to prevent paraplegia. They see the positive side effects: helping others and serving science. Other patients want to participate in this clinical study to support science, or rather progress. Research is necessary to advance and strengthen medicine.

Mrs. Weber: *And first of all,****it was for other people or you served science and you had to somehow.., that you advanced or to have new findings.****And I told myself “It can only be good for me”. I really have to say.*

(Mrs. Weber, 58, control group)

In order to “progress” or “serve science”, patients who participated in the MIS^2^ACE group talk about a certain “moral duty” to participate in a medical trial:

Mr. Neumann: I would. I would see it at some point as my moral duty to participate in [the trial]. Unless they tell you “I have to cut off a leg” just for fun, well not that. But if you agree on the risk somehow, and expect an added value in a case like that, so I would definitely participate in such a trial.

(Mr. Neumann, 43, MIS^2^ACE group)

In the case of Mrs. Weber, she is concerned about the fact that before randomisation she cannot know which treatment she will receive. Nevertheless, as well as perceiving a moral duty to participate in scientific investigation, she thinks of future generations.

Mrs. Weber: To get further in life you have to investigate and always theory. Somehow they need people saying „we go and …..try it out”

Interviewer: And what did that mean to you?

Mrs. Weber: Yes, to be honest. Because it works only, if it has not helped us or our generation or it went wrong but for our descendants it has only advantages, well that’s how I see it.

(Mrs. Weber, 58, control group)

Participants give diverse reasons for participation, but several have to do in general with generosity and the idea of “development of science”, but also with their interest in receiving better clinical care than might usually be expected.

## 4 The benefit and risks of invasive surgery

The patients in the PAPAartis study all receive one of two treatments for TAAA repair. The aim of treatment is to use a graft or stent to support the weak point in the artery and so eliminate the risk of its sudden rupture. However, the procedures entail risks as well as benefits. In the case of TAAA repair, the repair itself can induce paraplegia.

### 4.1 “Nothing worse than being a paraplegic”

Another reason for participating in the trial was the fear of suffering from paraplegia in the aftermath of the repair carried out with the conventional method. Some patients stated that the decision-making process was critical and they decided in favour of the trial expecting to lower the risk of paraplegia with the new method. The notion of suffering paraplegia as a result of the conventional repair method was accompanied by a variety of emotions of which fear stands out.

Mr. Neumann: ***"The fear of paraplegia is greater than the fear of dying"***

(Mr. Neumann, 43, MIS^2^ACE group)

Regarding the possibility of becoming paraplegic afterwards, some patients had in mind their socio-economic conditions they were living in:

Mrs. Weber: *We had the company’s Christmas party in December(…) and told (…) that there was a risk of paraplegia and that nobody has to be afraid because that is nothing for me [it would not concern me]!They had to laugh out loud because I.. I excluded [this option] for me from the beginning on. I have to say it as it is.****That is not an option for me. Although in the inside of me, I was afraid a bit****(…) My flat hasn’t enough space for a wheelchair, apart from that. But as I live on the first floor I had.. I was sitting at home and thought about a stair lift. And if what would be possible. But then I asked myself, “Why do you think about such crap?“.****That is not an option. Well but it concerns you, it really does.****[small break]. It is.. It does concern you though. But the risk exists. [breathing heavily]. (…) But if you have lost that [moving], there is no help anymore.*

(Mrs. Weber, 58, control group)

Regarding the emotions and expectations of the patients when they get a diagnosis of this magnitude, the fear of becoming a paraplegic is comparable with the fear of dying. The risk-benefit assessment of treatment is performed more or less consciously. Patients compare the possible consequences. The aim of the new intervention was to reduce the high risk of spinal cord injury associated with the conventional procedure, and the clinical study aims to provide evidence that it works. For some patients, the risk of spinal cord injury is perhaps even more frightening than the possibility that the aorta may suddenly rupture.

### 4.2 Disappointment at being allocated to the control group

Ms. Schröder's case is worth mentioning. Her initial hope of benefiting from the new method was not realized because she was randomized to the control group. Now Ms. Schröder weighs her options. She plays for time and monitors the growth of the aneurysm as observed by CT scans – waiting for the study to come to an end. She wants to see the results for herself. She hopes that enough patients will be found and that she herself will not be operated on as part of the control group. Her family background helps in decision-making - her son is a doctor. Their deliberations are due to their fear of paraplegia.

Mrs. Schröder: *My son is a physician and of course he always advises me. I always ask him and he always asks other colleagues. So he is always intensively involved and of course that always helps me with these decisions. So I was, I knew about this study very early on. And that it can be very positive. My son also said that. “Well, that sounds good and also seems reasonable. Why don't you take part in it?" And we thought that we could simply choose, eh the operation before the intervention. But Professor Frank said that was not possible. You have to take part in the study. That has not yet been proven [scientifically]. And that's why you have to take part in the study and then you'll, maybe you'll get the benefit [are lucky to join the new treatment] or you'll be in the control group. And that's why I was of course a bit disappointed that I'm now in the control group (…) I know I still have it ahead. But I had decided that we would postpone it a little longer. We had planned to have the operation in November. But then I had another CT scan done to record the stage and there was stagnation. That's why I would like to postpone the operation for a while, because it is also connected with a certain risk.*

Interviewer: *Yes, about the risk, this evaluating. Could you describe what it does with you?*

Mrs. Schröder: *Yes, the risk, that. I read the study documents and it said that every fifth to tenth patient suffers from paraplegia. And I thought that was actually a very high percentage (…) And then we did the CT to see if it was possible or if there was a dynamic in it. I have already had the upper part of the operation.*

(Mrs. Schröder, 72, control group)

### 4.3 The value of hope: from survival to realizing life's dreams

Quality of life is an important theme that recurs in the interviews. Patients talking about their recovery process mentioned how they wanted to go on and go back to what they were able to do prior to the surgery. Quality of life depends on their life's context and the conditions in which they live.

Interviewer: What do you enjoy?

Mrs. Meyer: that I’m still alive (…) and that God hasn’t got me ye*t*

(Mrs. Meyer, 79, MIS^2^ACE group)

Interviewer: Mrs. Bauer, how do you project yourself now for the near future?

Mrs. Bauer: That everything will be fine. That I'll be lively again like this, I mean, well, perhaps not as lively as I was before. But that I can go on living just normally.

you know?! that I'm not sitting in a wheelchair. For example! That's the most important thing for me!

(Mrs. Bauer, 65, control group)

In addition to reaching a certain “normality”, the hope of being cured opens up possibilities of fulfilling dreams, of improving the quality of life by accessing aspects of daily life such as traveling that were lost during the time of the disease.

Mr. Koch: ***I would like to get on my motorbike again with my girlfriend and we would go on beautiful bike- ehh motorbike tours again, yeah!***

(Mr. Koch, 63, MIS^2^ACE group)

## 5 Family context and care at home

### 5.1 The burden of illness on family life

In general, there is no doubt in any of our cases, that the family is an important issue for patients in the context of the trial, some appreciate family members' care work more than others. And in these cases, the family context affects the recovery process extremely. Additionally, in few cases the relationship between a patient and his or her partner has even changed over the time of the surgery. Above all, family plays an important role in the recovery and rehabilitation process after surgery.

Mrs. Fischer: *Yes it is [essential], in such a special situation when there are coming up diseases, for certain, well many compromise solutions, empathy from the one as from the other partner too. If you have to get through it and sometimes it leads to conflicts because it is such a difficult situation – not to mention the sexual love life – but the diseases over and over again, it is burdensome.****Both of them, the one with the disease even more probably, but the healthy one too in a certain manner, definitely. But as already said, it is how it is. You go through it together.***

(Mrs. Fischer, wife of Mr. Fischer, MIS^2^ACE group)

### 5.2 The desire for independence and control

In certain cases, female patients prefer to reject any kind of care service in order to maintain their independence. Although some female patients who participate in this research experience an extreme health situation, they find it difficult to accept help from the family environment or professional care, even though their state of health is very precarious. Gender roles and stereotypes appears as a key element in understanding illness suffering:

Interviewer: Did the doctor recommend a care service to come?

Mrs. Meyer: yeah, she did like that, I told her I don’t need one.. I have my medications, I know how to take it, as I have a list of those.

Interviewer: Would a care service be an option? For any reason or for any reason not?

Mrs. Meyer: No I don’t really need one.

Interviewer: okay

Mrs. Meyer: or anybody***!***

(Mrs. Meyer, 79, MIS^2^ACE group)

The will to lead an independent life is broken in the case of the only patient in our qualitative study who ended up being in a wheelchair. Mrs. Becker has no choice and needs help - she became a paraplegic because of the surgery, which was carried out with the conventional method and is now in fact disabled. The strong appreciation with regards towards her husband's care is expressed in her words:

Mrs. Becker: Well so, I have a slice of white bread for breakfast and for lunch I nearly eat normally. My husband cooks every day.

Interviewer: Your husband cooks for you? That means, you don’t have a service like “Meals on Wheels” or something similar?

Mrs. Becker: No. He cooks every day.

Interviewer: Okay.

Mrs. Becker: He is a sweetie isn’t he?!

(…)

They [family members] stopped by on their way home. And last weekend my son with his wife did the house cleaning. To help my husband out a bit (…) well so that he needn't do it all by himself. Cleaning the windows and so on (…)

yeah. And for my husband … sometimes it is too much!

(Mrs. Becker, 72, control group)

### 5.3 Adversity can sometimes strengthen family dynamics and relationships

The family context and care was a major influence on the patients' situation after the procedure. Some patients appreciate family members' care work more than others. As the following statements show, family helps in the recovery process. Additionally, in few cases the relationship between a patient and his or her partner has even changed over the time of the surgery, as Mrs. Klein explains:

Mrs. Klein: *You, You, how could I say it, you get closer together. You […] "I'll make myself a sandwich for supper now", "no I'm not eating yet". Now we have supper together again, now we sit down and take our time, we take more time for each other. We talk more. Than before. just this. well.(…) Well, it's no longer such an entrenched, routine thing.****Yes, it is now a bit more responsive to each other, more talking to each other and Yes, more being there for each other****and no longer "oh, I'm going to the garden now", "I. When shall we eat?", "yes, I'll come back sometime" […] now we eat at that time, and that is, […] I'm there. No,****the togetherness is again more intense let’s say (…) Especially because you realise now how quickly something like that can happen, can’t it?!***

(Mrs. Klein, 69, MIS^2^ACE group).

The family structure influences not only postsurgical care but also the patients will and strength to recover:

Mrs. Weber: *And so I thought, “it was destiny since they ascertained it and you will get through it”. That’s how I always was. And my grandchildren have always said, “granny, you will get better and we need you” and that is absolutely helping, isn’t it?*

(…) *it has.. I was. So first of all, my husband, and and and I have to say honestly, for the grandchildren, from my side, they have got me as the only grandma. They don’t have contact to the other granny, well and now losing me as the granny too. My son was really attached to my husband and even more to me. So there was …****they always said “you’ll get through this, you will get through this” and “we do that together”.****Never, it has never been an option to quit. Or to bury our heads in the sand. Or something like that. Never, that did never exist.*

(Mrs. Weber, 58, control group)

## 6 Treatment and recovery during a pandemic

### 6.1 Undergoing major surgery during a pandemic

The lack of contact with their relatives because of the restrictions caused by COVID-19 is a huge emotional problem for the patients. the lack of attention from loved ones has a major impact on their recovery process. For instance, Mrs Klein experiences the loneliness of being in a hospital stay without any visits from her family.

Mrs. Klein: *Yes, it was difficult. Because nobody could come. No one could come to Leipzig, to the hospital, to rehab. All that was still not allowed. So I had a telephone [call] later on. My sons, one lives in Gera, the other in Leipzig. Also my husband's sons […] They asked about me almost every day. My husband always passed on information of his own accord. And then there were very nice things, for example from my 10-year-old granddaughter.****“Grandma, we have to meet again soon. And you will get well very quickly. Remember […] who loves you.”***

(Mrs. Klein, 69, MIS^2^ACE group)

### 6.2 Being vulnerable to COVID-19

Patients are concerned about contracting COVID-19, on top of the process of recovering from the disease and treatment.

Yes, that's my plan. But of course that scares me too. Doesn’t it?! **Of course, then I really see myself in the very, very, very front row with pre-existing conditions.** Isn’t it?! It's not a great feeling. You know?! I deal with it somehow so that **I have the feeling that if it gets me, I'm dead, right?! Just this! My body, my lungs, will hardly be able to survive that.**

(Mr. Schäfer, did not want to divulge age, control group)

### 6.3 Cancelled appointments

In some cases, the operation schedule and the rehabilitation process is quite affected. Especially in the case of Mrs. Bauer, it has to be postponed:

Interviewer: Now regarding Hamburg, do you have another follow-up examination?

Mrs. Bauer: Yes, I was supposed to go there in June. But then I called right away and said, you can forget it.

Interviewer: Because of the crisis now, isn't it?

Mrs. Bauer: Yes, of course.

*(*Mrs. Bauer, MIS^2^ACE group*)*

## 7 Discussion

This study adds to a growing body of literature of qualitative research alongside clinical trials. Our results concur with other studies [1] that show the value of qualitative research in revealing the perspective of patients, gaining their confidence and addressing issues that might be too intimate to be discussed by other means.

Patients received copious information about the procedure of the trial and related documentation. They were given a 21-page description of the study due to the international requirements placed on clinical trials. Nevertheless, it is questionable if all of them were capable of understanding the documentation in order to make an informed decision whether to participate in the trial or not.

Our study suggested that limited scientific literacy was not, however, the only barrier to understanding. For many respondents, it was not simply that they did not fully understand the concepts of a control group or random allocation—they disliked and resisted them [9]. [8]suggested that information could be better tailored to the individual needs of the patients. For example [21], recommend producing an educational video to promote understanding of the aims of the study and increase recruitment. The PAPAartis trial did produce a video (PAPAartis 2020) although none of the interviewees mentioned that they had seen it, though one must note that it is only available in English.

Several participants stated the motivation of serving science or helping others, also mentioned in other literature [12,13]. This may be in part altruistic, though there may be an indirect benefit [22]. found that parents believe that their children may personally benefit in the future from the knowledge generated by the current research [12]. described the factor of altruism in the decision-making process as a by-product. Even if the patients felt they “had” to participate for other reasons, they also enjoyed being seen as an altruistic person. Nevertheless, as we do not have access to the patients who did not want to participate in the study, we cannot determine which factors are most important for participating. Trust in their clinician was an important factor in what decision was made.

As well as trusting the advice of the surgeons, many patients were able to articulate their own risk-benefit assessment. The patients decided whether to participate in the trial or not in order to get the most beneficial treatment for themselves. In the end, the decision-making process implied constantly balancing the pros and cons, as well keeping in mind the context in which they were living. Contrary to Ref. [23]; the participants of the PAPAartis study tried to avoid a possible consequence - being paraplegic - and not the treatment method. Many were convinced that the new intervention offers better odds of avoiding paraplegia, and most of the narratives confirmed that lowering the risk of becoming a paraplegic was the decisive factor in their decision to participate in the study. Some of the patients saw their quality of life in danger and worried about their future prospect of living in a wheelchair. Only one patient could point to a specific cause of the aneurysm. In this case, it was Marfan syndrome (a genetic disorder) and he recently found out that, in addition, his daughter and other family members suffer from the same syndrome.

As seen in Ref. [6]; family support was one of the most important components of recovery and wellbeing. As in Ref. [8]; our study also found the transition from hospital to home to be problematic for some patients. The degree of family support was crucial for a successful outcome and was in some cases severely affected by COVID-19 restrictions. Confirming the findings of [24]; the one patient who did suffer spinal cord injury stressed her appreciation of her partner and her desire never to give up [24]. Patients wished to be considered as individuals during their hospital care, and disapproved of “been seen as a number”. Going through cardiac surgery as in the PAPAartis trial, patients were concerned that they did not know what the future would bring, much the same as was described in Ref. [8]. A further similarity was that our patients also spoke in an admiring way of their doctors if they had told them honestly what to expect and what not. Several patients stated that they believed clinical care would be better if they were part of a clinical study (irrespective of their group allocation). Patients expressed a feeling of being “in good hands” or “taken care of”. In part, this was a question of trust in the clinician, though this belief does have some empirical foundation; there is evidence that patient outcomes are better in randomized control trials than in general practice [25].

The COVID-19 pandemic occurred early on in our study and we were able to adapt the research accordingly. Similar adaptations to research protocols have been made by other qualitative study teams [26,27]. While this certainly was not ideal, and some elements of the ethnographic process may have been lost, we did not find that remote interviewing presented an insurmountable challenge and, possibly, was more convenient for some patients. Other research teams reported other benefits of conducting interviews remotely, for example, by prompting the development of telehealth strategies [27],and some participants noted that participating in scientific study helped offset feelings of hopelessness during the pandemic [26].

The pandemic definitely was detrimental to the treatment and recovery process in hospital, as well as the transition back home. Above all, family visits were restricted or totally suspended, which affected the patients during their hospital stay as well as their recovery process at home. Moreover, some patients considered themselves as high-risk and were afraid of suffering COVID-19 in addition to feeling weak from the disease or in recovery from aneurysm repair [27]. focused on how the COVID-19 pandemic altered the treatment protocol in their study. In the PAPAartis trial we noticed similar disturbances due to the limitations caused by the pandemic. Patients felt insecure going home after treatment, and alone with their disease as personal contact is limited.

## 8 Conclusions

Our results show that patients participated in the clinical trial for different reasons: due to trust in doctors, the hope of having a better treatment, or for altruistic and collaborative reasons with science. However, many patients said they do not fully understand the information given to them. Avoidance of paraplegia played a fundamental role in the decision to participate in this trial. Family support and the socioeconomic conditions of the patients influenced the recovery process after surgery. The study has shown the impact of gender or other social determinants on health and recovery. Among the people interviewed, it seems that women are more affected by the need to continue maintaining care of others, despite their own health problems. The COVID-19 pandemic affected the healing process of the people who have participated in this research. Some elements of the ethnographic process may have been lost in the move to telephone interviews, though we have also shown that this can be convenient, feasible and acceptable to many participants. Qualitative research within the framework of a clinical trial opens the door to the inclusion of the experiences of patients in the processes of health, illness and disease, which allows understanding the impact of the clinical intervention on their quality of life.

## Declaration of competing interest

The authors declare that they have no known competing financial interests or personal relationships that could have appeared to influence the work reported in this paper.

## Data Availability

Data will be made available on request.

## References

[1] O'Cathain A. (2014). Getting added value from using qualitative research with randomized controlled trials: a qualitative interview study. Trials.

[2] Dismore L.L. (2019). What are the positive drivers and potential barriers to implementation of hospital at home selected by low-risk DECAF score in the UK: a qualitative study embedded within a randomised controlled trial. BMJ Open.

[3] Booth A. (2016). Guidance on choosing qualitative evidence synthesis methods for use in health technology assessments of complex interventions. https://www.researchgate.net/publication/298743768_Guidance_on_choosing_qualitative_evidence_synthesis_methods_for_use_in_health_technology_assessments_of_complex_interventions.

[4] Rapport F. (2013). Qualitative research within trials: developing a standard operating procedure for a clinical trials unit. Trials.

[5] Bradbury-Jones C. (2017). The state of qualitative research in health and social science literature: a focused mapping review and synthesis. Int. J. Soc. Res. Methodol..

[6] Patel S., Man W.D.C., Roberts N.J. (2020). Informal carers and peer support in pulmonary rehabilitation: an underutilized resource?. Curr. Opin. Support. Palliat. Care.

[7] Birtwistle S.B. (2021). Family support for physical activity post-myocardial infarction: a qualitative study exploring the perceptions of cardiac rehabilitation practitioners. Nurs. Health Sci..

[8] Doering L.V., McGuire A.W., Rourke D. (2002). Recovering from cardiac surgery: what patients want you to know. Am. J. Crit. Care.

[9] Naidoo N. (2020). The research burden of randomized controlled trial participation: a systematic thematic synthesis of qualitative evidence. BMC Med..

[10] Stead M. (2005). Hello, hello - it's English I speak!”: a qualitative exploration of patients' understanding of the science of clinical trials. J. Med. Ethics.

[11] Lie M. (2012). Let the computer choose?”: the experience of participants in a randomised preference trial of medical versus surgical termination of pregnancy. Sociol. Health Illness.

[12] Jackson C.J. (2010). Women's views and experiences of a patient preference trial in surgery: a qualitative study of the CARPET1 trial. Clin. Trials.

[13] Holmberg C. (2015). Gaining control over breast cancer risk: transforming vulnerability, uncertainty, and the future through clinical trial participation - a qualitative study. Sociol. Health Illness.

[14] Heaven B. (2006). Patients or research subjects? A qualitative study of participation in a randomised controlled trial of a complex intervention. Patient Educ. Counsel..

[15] Zoli S. (2010). Experimental two-stage simulated repair of extensive thoracoabdominal aneurysms reduces paraplegia risk. Ann. Thorac. Surg..

[16] Etz C.D. (2014). Spinal cord ischemia in open and endovascular thoracoabdominal aortic aneurysm repair: new concepts. J. Cardiovasc. Surg..

[17] Etz C.D. (2015). First-in-man endovascular preconditioning of the paraspinal collateral network by segmental artery coil embolization to prevent ischemic spinal cord injury. J. Thorac. Cardiovasc. Surg..

[18] Petroff D. (2019). Paraplegia prevention in aortic aneurysm repair by thoracoabdominal staging with “minimally invasive staged segmental artery coil embolisation” (MIS2ACE): trial protocol for a randomised controlled multicentre trial. BMJ Open.

[19] Urquhart C. (2012).

[20] Saunders B. (2018). Saturation in qualitative research: exploring its conceptualization and operationalization. Qual. Quantity.

[21] Barker R.E. (2020). The effects of a video intervention on posthospitalization pulmonary rehabilitation uptake. Am. J. Respir. Crit. Care Med..

[22] Drury N.E. (2021). Understanding parents' decision-making on participation in clinical trials in children's heart surgery: a qualitative study. BMJ Open.

[23] Armstrong N. (2016). Trial participation as avoidance strategy: a qualitative study. Health Expect..

[24] Angel S. (2010). Vulnerable, but strong: the spinal cord-injured patient during rehabilitation. Int. J. Qual. Stud. Health Well-Being.

[25] Martin-Ruiz E. (2018). Systematic review of the effect of adherenceto statin treatment on critical CardiovascularEvents and mortality in primary prevention. J. Cardiovasc. Pharmacol. Therapeut..

[26] Dunn S.L. (2021). Heart up! RCT protocol to increase physical activity in cardiac patients who report hopelessness: amended for the COVID-19 pandemic. Res. Nurs. Health.

[27] Lalande K. (2021). The healing hearts together randomized controlled trial and the COVID-19 pandemic: a tutorial for transitioning from an in-person to a web-based intervention. J. Med. Internet Res..

